# Association of LRP5 genotypes with osteoporosis in Tunisian post-menopausal women

**DOI:** 10.1186/1471-2474-15-144

**Published:** 2014-04-30

**Authors:** Rim Sassi, Hela Sahli, Chiraz Souissi, Hejer El Mahmoudi, Bechir Zouari, Amel Ben Ammar ElGaaied, Slaheddine Sellami, Serge Livio Ferrari

**Affiliations:** 1 Osteoporosis and Arthritis laboratory, Rabta hospital, Faculty of Medicine of Tunis, Tunis EL Manar University, Tunis, Tunisia; 2 Division of Bone Diseases, Rehabilitation and Geriatrics Department, Geneva University Hospital and Faculty of Medicine, Geneva, Switzerland; 3 Genetics, immunology and human pathologies Laboratory, Faculty of Mathematical, Physical and Natural Sciences of Tunis, Tunis EL Manar University, Tunis, Tunisia

**Keywords:** *LRP5*, Ala1330Val, Val667Met, Lumbar spine BMD, Fractures, Post-menopausal Tunisian population

## Abstract

**Background:**

Osteoporosis is a highly heritable trait. Among the genes associated with bone mineral density (BMD), the low-density lipoprotein receptor-related protein 5 gene (*LRP5*) has been consistently identified in Caucasians. However *LRP5* contribution to osteoporosis in populations of other ethnicities remains poorly known.

**Methods:**

To determine whether *LRP5* polymorphisms Ala1330Val and Val667Met are associated with BMD in North Africans, these genotypes were analyzed in 566 post-menopausal Tunisian women with mean age of 59.5 ± 7.7 years, of which 59.1% have low bone mass (T-score < −1 at spine or hip).

**Results:**

In post-menopausal Tunisian women, 1330Val was weakly associated with reduced BMD T-score at lumbar spine (p = 0.047) but not femur neck. Moreover, the TT/TC genotypes tended to be more frequent in women with osteopenia and osteoporosis than in women with normal BMD (p = 0.066). Adjusting for body size and other potential confounders, *LRP5* genotypes were no longer significantly associated with aBMD at any site.

**Conclusions:**

The less common Val667Met polymorphism showed no association with osteoporosis. The Ala1330Val polymorphism is weakly associated with lower lumbar spine bone density and osteopenia/osteoporosis in postmenopausal Tunisian women. These observations expand our knowledge about the contribution of *LRP5* genetic variation to osteoporosis risk in populations of diverse ethnic origin.

## Background

Osteoporosis is characterized by low bone mineral density (BMD) and micro architecture deterioration of bone tissue, leading to an increased risk of fractures that occur mostly at the hip, spine and wrist [1]. It is increasingly becoming a serious public-health issue because of the morbidity and mortality associated with the disease [2]. Hence, the identification and the management of women at highest risk of osteoporosis is a main concern.

Bone mineral density (BMD) is a primary predictor of osteoporotic fracture [3], and is used as a surrogate definition of osteoporosis [4]. BMD changes with age, peak levels being reached between the age of 20 and 30 and then decreasing during the later decades of life [5]. The bone mass acquired by adulthood varies from one population to another and is determined, essentially, genetically. In fact, genetic factors account for 50% to 80% of the interindividual variations in bone mineral density (BMD) [6-8].

To date, many genome-wide association studies (GWAS) were performed and multiple genes were identified to be associated with BMD and/or fragility fractures, including the low-density lipoprotein receptor-related protein 5 (*LRP5*) gene and other genes in the Wnt signalling pathway [9], like *CTNNB1*, and several genes in the RANKL/RANK signalling pathway, such as *TNFRSF11A*[10,11]. The Wnt signaling pathway has been shown to be related to bone mass and metabolism [12,13]. In osteoblasts, LRP5 can transduce canonical signals to promote renewal of stem cells, stimulation of pre-osteoblast replication, induction of osteoblastogenesis, and inhibition of osteoblast and osteocyte apoptosis by increasing the levels of ß-catenin and altering gene expression through the Lef/Tcf transcription factors [14]. It is known that LRP5 is essential for normal morphology, development processes, mechano-sensation and formation of bone [15]. Mapped on chromosome 11q12–14, activating mutations in the *LRP5* gene are responsible for rare conditions with a “high bone mass phenotype” [13,16] as well as autosomal dominant osteopetrosis type 1 [17]. Conversely, inactivating mutations are responsible for the osteoporosis pseudoglioma syndrome [12]. In addition to these mutations, a number of polymorphisms were described in the *LRP5* gene (http://www.ncbi.nlm.nih.gov/SNP). One of these, the Ala1330Val (rs3736228, exon 18) polymorphism, was associated with attenuated bone gain in prepubertal boys [18], decreased BMD in Japanese [19], American [20], Australian [21], and Dutch populations [22]. It was also associated with an increased risk of fragility fractures in postmenopausal Australian women [21] and in elderly Dutch men [22]. Similarly, the Val667Met (rs4988321, exon 9) polymorphism was associated with attenuated bone gain in prepubertal boys but not in girls participating in a 1-year longitudinal study [18]. In the same study, this polymorphism was also associated with decreased lumbar spine bone mineral content and projected bone area and there was a trend toward lower BMD in the spine [18].

The implication of Ala1330Val and Val667Met polymorphisms in BMD variation and risk of post-menopausal osteoporosis may differ between populations of various origins. However in North Africa, no association study was reported. To investigate whether allelic variations of the *LRP5* gene contribute to bone mass and osteoporosis in Tunisian population, we studied the association of Ala1330Val and Val667Met polymorphisms with BMD variation and osteoporosis risk in 566 Tunisian post-menopausal women.

## Methods

### Study population

Five hundred and sixty six postmenopausal Tunisian women, all with Tunisian ancestry, were recruited for osteoporosis consultation from the Rabta and Charles Nicolles Hospitals of Tunis. Women were prospectively recruited and included in the study regardless of their BMD values. Subjects with a history of bone disease, metabolic or endocrine diseases, hormone replacement therapy, and a corticotherpeutical history were excluded. Our study was approved by the National ethic committee and all participants provided written informed consent.

### Clinical measurements

The BMD was measured for the lumbar spine (L2-L4) and femoral neck (dual femur) by dual energy X-ray absorptiometry (DXA) using a GE-Lunar PRODIGYTM device.

The prevalence of clinical fractures was estimated by obtaining a fracture history for each subject including the age at the time of fracture, the site of fracture and the trauma intensity. Only fractures caused by low impact were included. All the characteristics of the studied population as age, anthropometric and obstetric parameters are shown in Table 1. The osteodensitometric results of our population are summarized in Table 2 and the classification of osteopenian and osteoporosis women were made according to T-SCORE < −1, respectively <2.5 at spine or hip.

**Table 1:** Characteristics of the studied Tunisian post menopausal women

**Variable**	**Mean ± SD**	**n**
Age (yr)	59.5 ± 7.7	
weight (kg)	72.0 ± 12.7	
BMI	28.9 ± 5.0	
Age at menarche (yr)	12.8 ± 1.6	
Age at menopause (yr)	48.4 ± 4.5	
Parity	3.4 ± 2.0	
Fracture		55
No fracture		511

**Table 2:** Mean BMD by WHO diagnostic group

**Mean ± SD**			
**Variable**	**Osteoporotic**	**Osteopenic**	**Normal**
	**n = 141**	**n = 194**	**n = 231**
**LS BMD (g/cm**^**2**^**)**	0.695 ± 0.065	0.827 ± 0.070	0.997 ± 0.092
**RFN BMD (g/cm**^**2**^**)**	0.769 ± 0.103	0.843 ± 0.096	0.971 ± 0.101
**LFN BMD (g/cm2)**	0.775 ± 0.096	0.837 ± 0.092	0.966 ± 0.113

LS, lumbar spine, RFN, right femur neck, LFN, left femur neck.

### SNP genotyping

Genomic DNA was extracted from blood by Phenol Chloroform procedure. Ala1330Val and Val667Met SNPs were genotyped by the pyrosequencing method, consisting of a series of four enzymatic steps resulting in sequencing a short region that include the SNP [23]. The specific primers used in the PCR are reported by Ferrari et al. study [18].

### Statistical analysis

Hardy-Weinberg equilibrium was tested for each SNP by use of the standard χ^2^ test. Data are represented as means ± SD. The categorical variables were compared between genotypes using the χ^2^ test. The associations between polymorphisms and BMD were assessed using Student *t* test. Due to the extremely low frequency (<10%) of the minor alleles, all analysis were performed under the assumption of a dominant model (combining homozygote for the minor allele and heterozygote in one group). Logistic regression models were applied, after controlling for several potential covariates (age, weight, height, menopause age, menarche age, parity and fractures). Association study and logistic regression were examined by SPSS software.

## Results

Our study comprised 566 Tunisian post-menopausal women with a mean age of 59.5 ± 7.7 years (Table 1). 59.1% had low BMD (24.9% osteoporotic and 34.1% osteopenic (Table 2). Among them, 55 women had a history of low trauma after the age of 45 years (Table 1). Val667Met is a G/A substitution in exon 9, with genotype frequencies GG: 88.0%, GA: 11.7%, and AA: 0.4%. Ala1330Val is a C/T substitution in exon18 with genotype frequencies CC: 79.9%, CT: 18.9%, and TT: 1.2%. The genotype frequencies of the 2 SNPs, studied in our population, did not significantly deviate from hardy Weinberg equilibrium (χ2 = 0.54 and 0.88 respectively for Val667Met and Ala1330Val). Association analysis are summarized in Table 3. A significant difference in LS BMD T-scores was found between CC and TC/TT genotypes at spine (p = 0.047), but not at hip. Moreover, the TT/TC genotypes tended to be more frequent in women with osteopenia and osteoporosis than in normal women (p = 0.066) (Table 3). In contrast, the Val667Met genotypes were not associated with aBMD and their distribution was similar between normal and osteoporotic/osteopenic women. Distribution of *LRP5* genotypes did not differ between women with and without fragility fractures (Table 3).

**Table 3:** **Association of LRP5 genotypes with BMD**^**a**^

**Polymorphisms**						
	**Val667Met**			**Ala1330Val**		
	***GG***	***GA + AA***	***P***	***CC***	***CT + TT***	***P***
**Parameter**	*n = 498*	*n = 68*	*value*^*b, e, F*^	*n = 452*	*n = 114*	*value*^*b*^
**Osteopenia**^**c**^	87.5	12.5		77.6	22.4	
**+osteoporosis (%)**^**d**^						
**Normals (%)**	88.7	11.3	0.373	83.1	16.9	0.066
**fractured women (%)**	89.1	10.9		85.5	14.5	
**Non fractured women (%)**	87.9	12.1	0.499	79.3	20.7	0.182
**BMD spine (g/cm2)**	0.869 ± 0.144	0.854 ± 0.134	0.438	0.873 ± 0.145	0.841 ± 0.134	0.072
**Tscore spine**	−1.085 ± 1.339	−1.327 ± 1.305	0.170	−1.044 ± 1.338	−1.388 ± 1.297	0.047*
**BMD right hip (g/cm2)**	0.880 ± 0.127	0.872 ± 0.136	0.62	0.882 ± 0.127	0.867 ± 0.131	0.359
**Tscore right hip**	−0.451 ± 1.066	−0.648 ± 1.225	0.176	−0.433 ± 1.060	−0.639 ± 1.183	0.164
**BMD left hip (g/cm2)**	0.877 ± 0.129	0.870 ± 0.127	0.685	0.879 ± 0.129	0.865 ± 0.127	0.448
**Tscore right hip**	−0.514 ± 1.019	−0.635 ± 1.131	0.380	−0.499 ± 0.999	−0.645 ± 1.155	0.350
**BMD total hip**	0.857 ± 0.120	0.851 ± 0.132	0.823	0.859 ± 0.120	0.847 ± 0.126	0.430
**Tscore total hip**	−0.652 ± 1.001	−0.801 ± 1.182	0.522	−0.635 ± 0.994	−0.807 ± 1.135	0.232

^a^Continuous variables (BMD values) are expressed as mean standard deviation, and categorical variables (osteopenia, osteoporosis, presence osteoporotical fracture) are expressed as percentages (%).

^b^Based on Student t test for continuous variables.

^c^World Health Organization definition: 1 standard deviatio ≥ T score ≤ 2.5 standard deviation (29).

^d^World Health Organization definition: T score ≤ 2.5 standard deviation (29).

^e^Based on χ2 test between two groups OP + O and Normales.

^f^Based on χ2 test between two groups fractured and non fractured women.

*p≤ 0,05 significant in bold character.

To further evaluate the independent contribution of *LRP5* polymorphisms to BMD in our population, we performed a logistic regression analysis including age, weight, age at menarche, age at menopause, parity, fractures and *LRP5* genotypes. As shown in Table 4, Age (p = 0.001) and weight (p = 0.0001), were the most significant contributors to BMD, followed by a non-significant contribution of parity (p = 0.073), and A1330Val genotypes (p = 0.11).

**Table 4:** Logistic regression analysis of spine

***Variable***	***B***	***t***	***P value***
***Age (yr)***	*−0.003*	*−3.471*	0.001*
***Weight (kg)***	*0.005*	*4.522*	***0.000***
***Age at menarche (yr)***	*−0.005*	*−1.71*	*0.942*
***Age at menopause (yr)***	*−0.002*	*−1.197*	*0.232*
***Parity***	*0.005*	*1.798*	*0.073*
***Fracture***	*−0.026*	*−1.333*	*0.183*
***Ala1330Val genotype***	*0.032*	*1.57*	*0.117*

*p≤ 0,05 significant in bold character.

## Discussion

For Tunisian women, a lumbar and femoral BMD analysis showed that their average values are close to those of the Asian and Caucasian women. At femoral neck, the lowest values were noted in European population. The osteoporosis prevalence was higher in Asian than in Tunisian and Caucasian population [24]. The differences observed between various populations can be caused by many environmental factors such as diet, physical activity, smoking alcohol and the geographical variability of vitamin D status. However, genetic determinism may also contribute to those differences.

During the past two decades, it has become clear that many genes contribute to the variation in BMD in the general population; however, candidate gene association studies have led to conflicting and contradictory findings [25]. Nevertheless, there is clear evidence for the association between genetic variation of *LRP5* and BMD in the general Caucasian and Chinese populations [18,19,21,26-30]. In African and North African populations, no information seems to be available about *LRP5* and BMD variation.

In our Tunisian post-menopausal women, *LRP5* polymorphisms frequencies were similar with previous studies reported in women with Caucasian background [22,31,32], but different from those observed in Asian population [19]. It should be noted that the association of the Ala1330Val variant with BMD and osteoporosis/osteopenia was in the same direction and had similar effect sizes in our study as in Europeans and Asians. However the absence of an association between BMD and the Val667Met variant in our population was distinct from previously consistent associations in other ethnic groups. This difference could represent a truly different genetic determination of osteoporosis risk between ethnicities or be due to a false negative result in our limited sample of Tunisian women, considering that the A allele is quite rare (6.1% < 10%).

Based on the physiological role of *LRP5* in bone biology, Val667Met and Ala1330Val should, in principle, result in decreased Wnt signaling. Val667Met resides at the top of the third propeller module in the extracellular domain of the receptor, a domain that is thought to interact with the Wnt-inhibitor Dkk1. Therefore, it is possible that the Val667Met mutant may have a higher binding affinity for Dkk1 as compared with the wild-type protein [33]. Ala1330Val is located within the second low-density lipoprotein (LDL) domain. Although the exact molecular mechanism remains to be clarified, the Ala1330Val mutant was shown to have a signicantly reduced Wnt-signaling capacity as compared with the wild type molecule in two independent TCF-Lef reporter assays [31,34].

The Ala1330Val seemed to be associated with lower spine bone density in post-menopausal Tunisian women. In fact, the TT/TC genotype could be more frequent in osteoporotic and osteopenic compared to women with normal BMD (p = 0.066). However the effect of this variant was detected only at the lumbar spine and not at the hip. Our results are concordant with those found in Asian and Caucasian populations. Thus Bich et al. [25] have demonstrated by a Bayesian meta-analysis that the A1330V variant was mainly associated with lumbar spine BMD in Asian populations, whereas in Caucasian populations associations occurred at both the lumbar spine and femoral neck [25,35,36]. The association of A1330V with lumbar spine than with femoral neck BMD is consistent with recent genome-wide association studies [11] and may reflect biological differences between the sites, a lower heritability for the femoral neck site [37,38], or higher measurement error at this site.

Richards et al. [11] noted that the *LRP5* polymorphisms were also significantly associated with fracture risk. But no significant difference was found between fractured and non-fractured women in our population. Considering the small number of fractured women in our sample (n = 55), our study is likely to be underpowered to detect such an association.

The logistic regression analysis for all the parameters including LRP5 genotypes reported a significant effect of age and weight, while the association with Ala1330Val genotypes was reduced. Since body size is strongly correlated with bone size, and therefore aBMD, this observation suggests that the association of *LRP5* polymorphisms with aBMD is at least partly mediated through LRP5 effects on bone size. This hypothesis is further supported by a previous study in Swiss young boys and girls in whom *LRP5* polymorphisms were associated with decreased lumbar spine bone mineral content (BMC) and projected bone area [18].

## Conclusions

In conclusions, the Ala1330Val variant seems to be weakly associated with lower lumbar spine BMD in Tunisian post-menopausal women, contributing to the notion that *LRP5* genetic variation is a ubiquitous determinant of osteoporosis risk among various ethnicities. More investigation about the interaction of *LRP5* polymorphisms and other factors notably hormonal, environmental and nutritional as well as physical activity are needed. These latter factors obviously vary from one individual to the next and from one population to the other, which can further influence the contribution of *LRP5* polymorphisms to BMD in diverse ethnic groups.

## Competing interests

We wish to confirm that there are no known competing interest associated with this publication and there has been no significant financial support for this work that could have influenced its outcome.

## Authors’ contributions

RS conceived and performed the molecular analysis and wrote the manuscript. HS is involved in clinical measurement. HEM and CS are involved in technical support and assistance. BZ performed statistical analysis. ABEG, SS and SLF designed the study. All authors read and approved the final manuscript.

## Pre-publication history

The pre-publication history for this paper can be accessed here:

http://www.biomedcentral.com/1471-2474/15/144/prepub

## References

[B1] Li WF Hou SX Yu B Li MM Férec C Chen JM Genetics of osteoporosis: accelerating pace in gene identification and validation Hum Genet 2010 127 3 249 285 10.1007/s00439-009-0773-z 20101412

[B2] Riggs BL Melton LJ The worldwide problem of osteoporosis: insights afforded by epidemiology Bone 1995 17 505 11 10.1016/8756-3282(95)00258-4 8573428

[B3] Nguyen T Sambrook P Kelly P Jones G Lord S Freund J Eisman J Prediction of osteoporotic fractures by postural instability and bone density BMJ 1993 307 6912 1111 1115 10.1136/bmj.307.6912.1111 8251809 PMC1679116

[B4] Kanis JA Glüer CC An update on the diagnosis and assessment of osteoporosis with densitometry. Committee of scientific advisors, international osteoporosis foundation Osteoporos Int 2000 11 3 192 202 10.1007/s001980050281 10824234

[B5] Kanis JA Melton LJ 3rd Christiansen C Johnston CC Khaltaev N The diagnosis of osteoporosis J Bone Miner Res 1994 9 8 1137 41 7976495 10.1002/jbmr.5650090802

[B6] Williams FM Acta Reumatol Port 2007 32 231 240 17940498

[B7] Ralston SH Genetics of osteoporosis Proc Nutr Soc 2007 66 2 158 65 10.1017/S002966510700540X 17466098

[B8] Pocock NA Eisman JA Hopper JL Yeates MG Sambrook PN Eberl S Genetic determinants of bone mass in adults. A twin study J Clin Invest 1987 80 3 706 710 10.1172/JCI113125 3624485 PMC442294

[B9] Johnson ML Harnish K Nusse R Van Hul W LRP5 and Wnt signaling: a union made for bone J Bone Miner Res 2004 19 11 1749 1757 10.1359/JBMR.040816 15476573

[B10] Rivadeneira F Styrkársdottir U Estrada K Halldórsson BV Hsu YH Richards JB Zillikens MC Kavvoura FK Amin N Aulchenko YS Cupples LA Deloukas P Demissie S Grundberg E Hofman A Kong A Karasik D van Meurs JB Oostra B Pastinen T Pols HA Sigurdsson G Soranzo N Thorleifsson G Thorsteinsdottir U Williams FM Wilson SG Zhou Y Ralston SH van Duijn CM Genetic factors for osteoporosis (GEFOS) consortium. Twenty bone-mineral-density loci identified by large-scale meta-analysis of genome-wide association studies Nat Genet 2009 41 11 1199 1206 10.1038/ng.446 19801982 PMC2783489

[B11] Richards JB Kavvoura FK Rivadeneira F Styrkársdóttir U Estrada K Halldórsson BV Hsu YH Zillikens MC Wilson SG Mullin BH Amin N Aulchenko YS Cupples LA Deloukas P Demissie S Hofman A Kong A Karasik D van Meurs JB Oostra BA Pols HA Sigurdsson G Thorsteinsdottir U Soranzo N Williams FM Zhou Y Ralston SH Thorleifsson G van Duijn CM Kiel DP Genetic factors for osteoporosis consortium. Collaborative meta-analysis: associations of 150 candidate genes with osteoporosis and osteoporotic fracture Ann Intern Med 2009 151 8 528 537 10.7326/0003-4819-151-8-200910200-00006 19841454 PMC2842981

[B12] Gong Y Slee RB Fukai N Rawadi G Roman-Roman S Reginato AM Wang H Cundy T Glorieux FH Lev D Zacharin M Oexle K Marcelino J Suwairi W Heeger S Sabatakos G Apte S Adkins WN Allgrove J Arslan-Kirchner M Batch JA Beighton P Black GC Boles RG Boon LM Borrone C Brunner HG Carle GF Dallapiccola B De Paepe A LDL receptor-related protein 5 (LRP5) affects bone accrual and eye development Cell 2001 107 4 513 523 10.1016/S0092-8674(01)00571-2 11719191

[B13] Little RD Carulli JP Del Mastro RG Dupuis J Osborne M Folz C Manning SP Swain PM Zhao SC Eustace B Lappe MM Spitzer L Zweier S Braunschweiger K Benchekroun Y Hu X Dair R Chee L FitzGerald MG Tulig C Caruso A Tzellas N Bawa A Franklin B McGuire S Nogues X Gong G Allen KM Anisowicz A Morales AJ A mutation in the LDL receptor-related protein 5 gene results in the autosomal dominant high-bone-mass trait Am J Hum Genet 2002 70 11 19 10.1086/338450 11741193 PMC419982

[B14] Krishnan V Bryant HU Macdougald OA Regulation of bone mass by Wnt signaling J Clin Invest 2006 116 5 1202 1209 10.1172/JCI28551 16670761 PMC1451219

[B15] Li X Zhang Y Kang H Liu W Liu P Zhang J Harris SE Wu D Sclerostin binds to LRP5/6 and antagonizes canonical Wnt signaling J Biol Chem 2005 280 19883 19887 10.1074/jbc.M413274200 15778503

[B16] Boyden LM Mao J Belsky J Mitzner L Farhi A Mitnick MA Wu D Insogna K Lifton RP High bone density due to a mutation in LDL-receptor-related protein 5 N Engl J Med 2002 346 20 1513 1521 10.1056/NEJMoa013444 12015390

[B17] Van Wesenbeeck L Cleiren E Gram J Beals RK Bénichou O Scopelliti D Key L Renton T Bartels C Gong Y Warman ML De Vernejoul MC Bollerslev J Van Hul W Six novel missense mutations in the LDL receptor-related protein 5 (LRP5) gene in different conditions with an increased bone density Am J Hum Genet 2003 72 3 763 771 10.1086/368277 12579474 PMC1180253

[B18] Ferrari SL Deutsch S Choudhury U Chevalley T Bonjour JP Dermitzakis ET Rizzoli R Antonarakis SE Polymorphisms in the low-density lipoprotein receptor-related protein 5 (LRP5) gene are associated with variation in vertebral bone mass, vertebral bone size, and stature in whites Am J Hum Genet 2004 74 866 875 10.1086/420771 15077203 PMC1181981

[B19] Mizuguchi T Furuta I Watanabe Y Tsukamoto K Tomita H Tsujihata M Ohta T Kishino T Matsumoto N Minakami H Niikawa N Yoshiura K LRP5, low-density-lipoproteinreceptor- related protein 5, is a determinant for bone mineral density J Hum Genet 2004 49 80 86 10.1007/s10038-003-0111-6 14727154

[B20] Koller DL Ichikawa S Johnson ML Lai D Xuei X Edenberg HJ Conneally PM Hui SL Johnston CC Peacock M Foroud T Econs MJ Contribution of the LRP5 gene to normal variation in peak BMD in women J Bone Miner Res 2005 20 75 80 10.1359/jbmr.2005.20.1.75 15619672

[B21] Bollerslev J Wilson SG Dick IM Islam FM Ueland T Palmer L Devine A Prince RL LRP5 gene polymorphisms predict bone mass and incident fractures in elderly Australian women Bone 2005 36 599 606 10.1016/j.bone.2005.01.006 15777745

[B22] Van Meurs JB Rivadeneira F Jhamai M Hugens W Hofman A van Leeuwen JP Pols HA Uitterlinden AG Common genetic variation of the low-density lipoprotein receptor-related protein 5 and 6 genes determines fracture risk in elderly white men J Bone Miner Res 2006 21 141 150 16355283 10.1359/JBMR.050904

[B23] Ronaghi M Pyrosequencing sheds light on DNA sequencing Genome Res 2001 11 1 3 11 10.1101/gr.11.1.3 11156611

[B24] Sahli H Testouri N Chihaoui MB Salah AH Cheour E Meddeb N Zouari B Sellami S Bone mineral density in healthy Tunisian women Maturitas 2009 63 3 227 232 10.1016/j.maturitas.2009.03.014 19398172

[B25] Tran BNH Nguyen ND Eisman JA Nguyen TV Association between LRP5 polymorphism and bone mineral density: a Bayesian meta-analysis BMC Medical Genetics 2008 9 55 doi:10.1186/1471-2350-9-55, 2008 18588671 10.1186/1471-2350-9-55 PMC2459152

[B26] Brixen K Beckers S Peeters A Piters E Balemans W Nielsen TL Wraae K Bathum L Brasen C Hagen C Andersen M Van Hul W Abrahamsen B Polymorphisms in the low-density lipoprotein receptor-related protein 5 (LRP5) gene are associated with peak bone mass in non-sedentary men: results from the Odense androgen study Calcif Tissue Int 2007 81 421 429 10.1007/s00223-007-9088-z 18058054 PMC2151961

[B27] Giroux S Elfassihi L Cardinal G LaXamme N Rousseau F LRP5 coding polymorphisms inXuence the variation of peak bone mass in a normal population of French–Canadian women Bone 2007 40 1299 1307 10.1016/j.bone.2007.01.004 17307038

[B28] Koay MA Woon PY Zhang Y Miles LJ Duncan EL Ralston SH Compston JE Cooper C Keen R Langdahl BL MacLelland A O’Riordan J Pols HA Reid DM Uitterlinden AG Wass JA Brown MA InXuence of LRP5 polymorphisms on normal variation in BMD J Bone Miner Res 2004 19 1619 1627 10.1359/JBMR.040704 15355556

[B29] Urano T Shiraki M Ezura Y Fujita M Sekine E Hoshino S Hosoi T Orimo H Emi M Ouchi Y Inoue S Association of a single- nucleotide polymorphism in low-density lipoprotein receptorrelated protein 5 gene with bone mineral density J Bone Miner Metab 2004 22 341 345 15221492 10.1007/s00774-003-0492-9

[B30] Zhang ZL Qin YJ He JW Huang QR Li M Hu YQ Liu YJ Association of polymorphisms in low-density lipoprotein receptor- related protein 5 gene with bone mineral density in postmenopausal Chinese women Acta Pharmacol Sin 2005 26 1111 1116 10.1111/j.1745-7254.2005.00173.x 16115379

[B31] Kiel DP Ferrari SL Cupples LA Karasik D Manen D Imamovic A Herbert AG Dupuis J Genetic variation at the low-density lipoprotein receptor-related protein 5 (LRP5) locus modulates Wnt signaling and the relationship of physical activity with bone mineral density in men Bone 2007 40 587 596 10.1016/j.bone.2006.09.029 17137849 PMC1845172

[B32] Xiong DH Lei SF Yang F Wang L Peng YM Wang W Recker RR Deng HW Low-density lipoprotein receptor-related protein 5 (LRP5) gene polymorphisms are associated with bone mass in both Chinese and whites J Bone Miner Res 2007 22 385 393 10.1359/jbmr.061116 17241106

[B33] Van Meurs JB Trikalinos TA Ralston SH Balcells S Brandi ML Brixen K Kiel DP Langdahl BL Lips P Ljunggren O Lorenc R Obermayer-Pietsch B Ohlsson C Pettersson U Reid DM Rousseau F Scollen S Van Hul W Agueda L Akesson K Benevolenskaya LI Ferrari SL Hallmans G Hofman A Husted LB Kruk M Kaptoge S Karasik D Karlsson MK Lorentzon M Large-scale analysis of association between LRP5 and LRP6 variants and osteoporosis JAMA 2008 299 1277 1290 10.1001/jama.299.11.1277 18349089 PMC3282142

[B34] Urano T Shiraki M Usui T Sasaki N Ouchi Y Inoue S A1330 V variant of the low-density lipoprotein receptor-related protein 5 (LRP5) gene decreases Wnt signaling and aVects the total body bone mineral density in Japanese women Endocr J 2009 56 625 631 10.1507/endocrj.K09E-133 19571442

[B35] Lee YH Woo JH Choi SJ Ji JD Song GG Association between the A1330V polymorphism of the low-density lipoprotein receptor-related protein 5 gene and bone mineral density: a meta-analysis Rheumatol Int 2009 29 5 539 544 10.1007/s00296-008-0745-y 18932002

[B36] Liu JM Zhang MJ Zhao L Cui B Li ZB Zhao HY Sun LH Tao B Li M Ning G Analysis of recently identified osteoporosis susceptibility genes in Han Chinese women J Clin Endocrinol Metab 2010 95 9 E112 120 10.1210/jc.2009-2768 20554715

[B37] Karasik D Myers RH Cupples LA Hannan MT Gagnon DR Herbert A Kiel DP Genome screen for quantitative trait loci contributing to normal variation in bone mineral density: the Framingham Study J Bone Miner Res 2002 17 1718 1727 10.1359/jbmr.2002.17.9.1718 12211443

[B38] Stathopoulou MG Dedoussis GV Trovas G Katsalira A Hammond N Deloukas P Lyritis GP Low-density lipoprotein receptor-related protein 5 polymorphisms are associated with bone mineral density in Greek postmenopausal women: an interaction with calcium intake J Am Diet Assoc 2010 110 7 1078 1083 10.1016/j.jada.2010.04.007 20630166

